# SARS-CoV-2 B.1.1.7 UK Variant of Concern Lineage-Related Perceptions, COVID-19 Vaccine Acceptance and Travel Worry Among Healthcare Workers

**DOI:** 10.3389/fpubh.2021.686958

**Published:** 2021-05-26

**Authors:** Mohamad-Hani Temsah, Mazin Barry, Fadi Aljamaan, Abdullah N. Alhuzaimi, Ayman Al-Eyadhy, Basema Saddik, Fahad Alsohime, Ali Alhaboob, Khalid Alhasan, Ali Alaraj, Rabih Halwani, Amr Jamal, Nurah Alamro, Reem Temsah, Samia Esmaeil, Shuliweeh Alenezi, Fahad Alzamil, Ali M. Somily, Jaffar A. Al-Tawfiq

**Affiliations:** ^1^Pediatric Department, College of Medicine, King Saud University, Riyadh, Saudi Arabia; ^2^Division of Infectious Diseases, Department of Internal Medicine, College of Medicine, King Saud University and King Saud University Medical City, Riyadh, Saudi Arabia; ^3^College of Medicine, King Saud University, Riyadh, Saudi Arabia; ^4^Critical Care Department, College of Medicine, King Saud University, Riyadh, Saudi Arabia; ^5^Division of Pediatric Cardiology, Department of Cardiac Sciences, College of Medicine, King Saud University, Riyadh, Saudi Arabia; ^6^Heart Center, King Faisal Specialist Hospital & Research Center, Riyadh, Saudi Arabia; ^7^Sharjah Institute of Medical Research, University of Sharjah, Sharjah, United Arab Emirates; ^8^Department of Community and Family Medicine, College of Medicine, University of Sharjah, Sharjah, United Arab Emirates; ^9^Dr Sulaiman Al Habib Medical Group, Riyadh, Saudi Arabia; ^10^Department of Medicine, College of Medicine, Qassim University, Qassim, Saudi Arabia; ^11^Department of Clinical Sciences, College of Medicine, University of Sharjah, Sharjah, United Arab Emirates; ^12^Department of Family and Community Medicine, King Saud University Medical City, Riyadh, Saudi Arabia; ^13^Evidence-Based Health Care & Knowledge Translation Research Chair, King Saud University, Riyadh, Saudi Arabia; ^14^College of Pharmacy, Alfaisal University, Riyadh, Saudi Arabia; ^15^Department of Psychiatry, College of Medicine, King Saud University, Riyadh, Saudi Arabia; ^16^Department of Pathology and Laboratory Medicine, College of Medicine, King Saud University and King Saud University Medical City, Riyadh, Saudi Arabia; ^17^Specialty Internal Medicine and Quality Department, Johns Hopkins Aramco Healthcare, Dhahran, Saudi Arabia; ^18^Infectious Disease Division, Department of Medicine, Indiana University School of Medicine, Indianapolis, IN, United States; ^19^Infectious Disease Division, Department of Medicine, Johns Hopkins University School of Medicine, Baltimore, MD, United States

**Keywords:** COVID-19 vaccine, B.1.1.7 variant, heathcare workers perception, travel worry, COVID-19 vaccine acceptance, UK variant of concern

## Abstract

**Background:** Healthcare workers' (HCWs') travel-related anxiety needs to be assessed in light of the emergence of SARS-CoV-2 mutations.

**Methods:** An online, cross-sectional questionnaire among HCWs between December 21, 2020 to January 7, 2021. The outcome variables were HCWs' knowledge and awareness of the SARS-CoV-2 B.1.1.7 lineage that was recently reported as the UK variant of concern, and its associated travel worry and Generalized Anxiety Disorder (GAD-7) score.

**Results:** A total of 1,058 HCWs completed the survey; 66.5% were female, 59.0% were nurses. 9.0% indicated they had been previously diagnosed with COVID-19. Regarding the B.1.1.7 lineage, almost all (97.3%) were aware of its emergence, 73.8% were aware that it is more infectious, 78.0% thought it causes more severe disease, and only 50.0% knew that current COVID-19 vaccines are effective in preventing it. Despite this, 66.7% of HCWs were not registered to receive the vaccine. HCWs' most common source of information about the new variant was social media platforms (67.0%), and this subgroup was significantly more worried about traveling. Nurses were more worried than physicians (*P* = 0.001).

**Conclusions:** Most HCWs were aware of the emergence of the SARS-CoV-2 B.1.1.7 variant and expressed substantial travel worries. Increased worry levels were found among HCWs who used social media as their main source of information, those with lower levels of COVID-19 vaccine uptake, and those with higher GAD-7 scores. The utilization of official social media platforms could improve accurate information dissemination among HCWs regarding the Pandemic's evolving mutations. Targeted vaccine campaigns are warranted to assure HCWs about the efficacy of COVID-19 vaccines toward SARS-CoV-2 variants.

## Introduction

The emergence of the Severe Acute Respiratory Syndrome Coronavirus 2 (SARS-CoV-2) has resulted in a global pandemic. Being an RNA virus, SARS-CoV-2 can mutate over time, and thus multiple SARS-CoV-2 variants are circulating globally (1). As of December 13, 2020, there were 1,108 reported cases of people infected with the B.1.1.7 variant in the United Kingdom (UK) in almost 60 different local authorities, with the exact number likely much higher (2). The UK variant under investigation in this study was discovered in December 2020 (VUI-202012/01) and is characterized by a set of 17 mutations (2). One of its most significant mutations is N501Y in the gene coding of the spike protein, which attaches to the angiotensin-converting enzyme 2 receptors (2).

The B.1.1.7 lineage was first identified by the COVID-19 Genomics UK consortium, which performs random genetic sequencing of positive COVID-19 samples in the UK. Since its creation in April 2020, the consortium has sequenced 140,000 virus genomes from individuals infected with COVID-19 and publishes weekly reports on its website (3). This B.1.1.7 variant is estimated to have first emerged in the UK in September 2020 (1). As the variant rapidly spread in the subsequent few weeks, by December 26, 2020, more than 3,000 cases of this new variant have been reported in the UK (4).

This mutation has been found to have a high transmission rate and has become one of the main circulating variants in several locations in the UK (2). This variant has since been detected in many other countries around the world, including the United States (U.S.) and Canada (1). The European Centre for Disease Prevention and Control (ECDC) recommends that residents of the most affected areas restrict movement and travel, including international travel outside of these areas (4). Several countries, including the UK, have imposed travel restrictions to limit the variant's rapid spread (4).

Scientists and researchers have begun to learn more about this variant to better understand its virulence and infectiousness and whether currently authorized COVID-19 vaccines would illicit immunity against it (1). The rapid spread of B.1.1.7 has prompted the ECDC to announce that the overall risk associated with the introduction and further spread of this SARS-CoV-2 strain is high (4). Despite the current lack of evidence that these variants may cause a more severe illness or increased risk of death, healthcare workers (HCWs) are yet again challenged by a new stressful situation that could affect their psychological well-being and/or travel arrangements (1, 5).

The Kingdom of Saudi Arabia (KSA) took multiple steps to decrease the spread of COVID-19, including the following: suspending the country's eVisa program and placing a ban on inbound travel, including the neighboring Gulf countries. In addition, on March 7, 2020, KSA limited international flights to the three major airports within the Kingdom and required a negative PCR test for SARS-CoV-2 for arriving travelers (6). The rapid spread of this new strain has prompted stricter international travel bans to and from affected countries (from December 21, 2020, to January 7, 2021). The closure of airports and restrictions to travel are likely to cause panic and concerns, particularly among healthcare workers, where two-third of healthcare workers in KSA are expatriates (7). To date, all international flights remain very limited to essential workers such as healthcare workers. Media outlets have announced that many non-Saudi expat HCWs have been stranded abroad and are still struggling to return to the KSA, with available flights in short supply (8). The emergence of the new strain is expected to introduce further restrictions making travel even more difficult, thus potentially causing anxiety and concerns among this group. Also, baseline higher anxiety levels were reported to be more common in medical personal, especially female doctors and nurses (9). Starting on December 17, 2020, Saudi Arabia has rolled out mass BNT162b2 vaccinations for HCWs, with open registration beginning a week earlier (10). As the rapid evolution of the situation warrants further research, we conducted this study among HCWs in Saudi Arabia to assess their perceptions, anxiety levels, and travel worries caused by this new SARS-COV-2 variant. We hypothesized that the presence of underlying generalized anxiety, lack of awareness of new B.1.1.7 variant, and personal risk factors for severe COVID-19 infections (e.g., older age) might contribute to increased worry level of travel.

In this study, we examined the awareness HCWs in Saudi Arabia of the new B.1.1.7 SARS-COV-2 variant, utilizing several variables that may contribute to their awareness of the new variant spreading across international borders, imposing additional risks upon international travel, and ultimately imparting to the fear and anxiety surrounding travel and its recent restrictions. The variables assessed in our study included gender, age, nationality, level of education, and profession. Moreover, we attempted to link these factors to the level of anxiety, travel perception, personal and professional experience of dealing with COVID-19, and the source of knowledge.

## Method

### Data Collection

This multicenter, cross-sectional survey was conducted among HCWs in Saudi Arabia during the COVID-19 pandemic. Data were collected between December 27, 2020 and January 3, 2021. At the time of data collection, there were at least a handful of countries that had reported infection with the B.1.1.7 variant of SARS-CoV-2. HCWs were surveyed regarding their perceptions, anxiety levels, and travel worry caused by the new variant. Participants were invited using a convenience sampling technique. We used several social media platforms and email lists to recruit participants. The survey was a pilot-validated, self-administered questionnaire that was sent to HCWs online through SurveyMonkey^©^, a platform that allows researchers to deploy and analyze surveys via the web. The questionnaire was adapted from our previously published study with modification and additions related to the new SARS-COV-2 variant (11–13).

The questions asked about respondents' demographic characteristics (job category, age, sex, years of clinical experience, and work area), previous exposure to COVID-19 patients, travel history in the previous 3 months, and whether they had received and/or registered to receive the COVID-19 vaccine. We assessed the following outcomes related to the new SARS-COV-2 variant: knowledge, perceptions, and travel worries. In addition, we assessed factors affecting respondents' worry level regarding international travel as well as HCWs' sources of information about the B.1.1.7 SARS-CoV-2 mutant variant. HCWs' anxiety was also measured by the validated 7-item General Anxiety Disorder (GAD-7) questionnaire, which has been used in several studies assessing HCWs' anxiety levels during the pandemic (12, 14).

HCWs were informed of the purpose of the study in English at the beginning of the online survey. The respondents were given the opportunity to ask questions via a dedicated email address for the study. The Institutional Review Board at the College of Medicine and King Saud University Medical City approved the study (approval #20/0065/IRB). A waiver for signed consent was obtained since the survey presented no more than a minimal risk to subjects and involved no procedures for which written consent is usually required. To maximize confidentiality, personal identifiers were not required.

### Statistical Analyses

Descriptive analyses using means and standard deviations were applied to continuously measured variables, and categorically measured variables were described with frequencies and percentages. The histogram and the statistical Kolmogorov–Smirnov tests of normality were used to assess the statistical normality of the measured continuous variables. The multiple response dichotomy analysis was applied to the multiple option questions, such as the one asking about sources of information. Respondents' awareness of the new mutagenic SARS-COV-2 virus strain was measured with eight questions, which received a score of 1 for each correctly answered knowledge/awareness question and 0 for incorrectly answered questions. A total awareness of mutagenic viral outbreak score was measured by adding the scores for the knowledge indicators (range: 0–8 points). For categorically measured variables, the independent samples *t*-test and one-way ANOVA test were used to assess the statistical significance of HCWs' mean perceived worry regarding travel. Pearson's bivariate test of correlation was used to assess the correlations between metric variables. Multivariate linear regression analysis was used to assess the multivariate associations of HCWs' demographic and professional characteristics and their perceptions with their worry level about traveling abroad.

The collinearity & multicollinearity between independent variables were tested using bivariate correlations, variance inflation indices factor (VIF), and tolerance statistics. These parameters were within an acceptable range. IBM SPSS Statistics for Windows, Version 21.0. Armonk, NY: IBM Corp. was used for the statistical data analysis, and results were considered significant at the 0.05 level.

## Results

In total, 1,212 HCWs participated in the survey. Of these, 1,058 completed the survey, resulting in a completion rate of 87.2%. Most participants were female (66.5%), between the ages of 31 and 40 years (44.5%) and married (75.7%). Most respondents were nurses (59.2%), followed by physicians (38.0%). Participants worked in intensive care units (ICUs) (25.8%), general wards (24.7%), or outpatient departments (OPDs) (21.3%) in various hospital settings (Table 1).

**Table 1:** Descriptive analysis of HCWs' sociodemographic and professional characteristics (*N* = 1,058).

**Characteristic**	***n***	**%**
**Sex**		
Male	354	33.5
Female	704	66.5
**Age**		
20–30 years	238	22.5
31–40 years	471	44.5
41–50 years	263	24.9
≥ 51 years	86	8.1
**Marital status**		
Single	257	24.3
Married	801	75.7
**Nationality**		
Expatriate	736	69.6
Saudi	322	30.4
**Clinical role**		
Consultant	213	20.1
Assistant consultant/fellow	52	4.9
Resident/registrar	138	13.0
Nurse	626	59.2
Intern/medical student	29	2.7
**Hospital ward**		
ICU	273	25.8
ER	110	10.4
OR	62	5.9
Isolation ward	63	6.0
General ward	261	24.7
OPD	225	21.3
Non-clinical area	64	6.0
**Hospital sector**		
Private	174	16.4
Public/governmental	487	46.0
University hospital	397	37.5
**Hospital specialty**		
Primary healthcare center	123	11.6
Secondary care hospital	196	18.5
Tertiary hospital	739	69.8

*ICU, intensive care unit; ER, emergency room; OR, operating room; OPD, outpatient department*.

The majority of HCWs (67.9%) reported managing COVID-19 patients. Additionally, 22.8% had encountered an infected family member, and 9.1% reported having been infected with laboratory-confirmed COVID-19 themselves. Only (11.9%) have already received the first dose of the vaccine and another (21.4%) had registered the COVID-19 vaccine. Almost all HCWs reported that they were aware of the B.1.1.7 variant (97.3%), with 36.0% reporting that they had sufficient knowledge of the variant. Although most participants (64.3%) were unsure whether the new mutation could cause a false-negative polymerase chain reaction (PCR) result, 18.9% of them thought it would. Most of the respondents (59.8%) agreed that the management of the new mutation would be similar to the current COVID-19 management guidelines. More than half of the participants (53.9%) believed that the new SARS-CoV-2 mutation outbreak in foreign countries would result in subsequent lockdowns if it reached the KSA. When HCWs were asked to indicate their level of agreement on the need to impose tighter infection control measures due to the new mutation variant, a high level of agreement (M = 4.08 on a scale of 5, SD = 1.05) was found. Regarding worry about international travel within the next month, high levels of worry were found among participants, with the highest levels of the worry associated with travel to the UK (M = 3.25, SD = 1.35), which was slightly higher than their worry levels about traveling abroad generally (M = 3.22, SD = 1.07) (Table 2).

**Table 2:** HCWs' perceptions and experiences of the COVID-19 pandemic and its mutations.

**Question**	**Answer**	***n***	**%**
Have you managed COVID-19 patients?	No	340	32.1
	Yes	718	67.9
Have any of your family members been infected with COVID-19?	No	817	77.2
	Yes	241	22.8
Have you been previously diagnosed with PCR-confirmed COVID-19?	No	962	90.9
	Yes	96	9.1
Have you traveled abroad in the last 3 months?	No	998	94.3
	Yes	60	5.7
Have you received or registered to receive the COVID-19 vaccine?	Yes: I received the first dose of the COVID vaccine	126	11.9
	Yes: I have registered but have not received it yet	226	21.4
	No	706	66.7
Do you know about the new SARS-CoV-2 B.1.1.7 variant that was reported internationally this month?	Yes: I am knowledgeable about this topic	381	36.0
	Yes: but I have limited knowledge	649	61.3
	No	28	2.6
The COVID-19 mutation may/will result in another local lockdown in Saudi Arabia if it causes local outbreaks.	Agree	570	53.9
	Neither agree nor disagree	380	35.9
	Disagree	108	10.2
The mutation MAY cause a false NEGATIVE PCR result.	Agree	200	18.9
	Neither agree nor disagree	680	64.3
	Disagree	178	16.8
**Question**	**Answer**	**Mean (SD)**	
With respect to the mutation, how much do you agree that healthcare workers should apply tighter infection control measures?	1: Strongly disagree 2: Disagree 3: Neutral 4: Agree 5: Strongly agree	4.08 (1.05)	
In relation to the newly reported COVID-19 mutation, how worried are you about international travel?	1: Not at all worried 2: Slightly worried 3: Moderately worried 4: Very worried 5: Extremely worried	3.22 (1.17)	
How worried would you be to travel to the following countries in the next month?^*^	The UK	3.25 (1.35)	
	Other European nations	3.10 (1.32)	
	Other world destinations outside of Europe and the UK	2.74 (1.23)	

* *This question uses the same scale as the previous question*.

### HCWs' Awareness of and Sources of Information About the New COVID-19 Mutation

**Figure 1:**
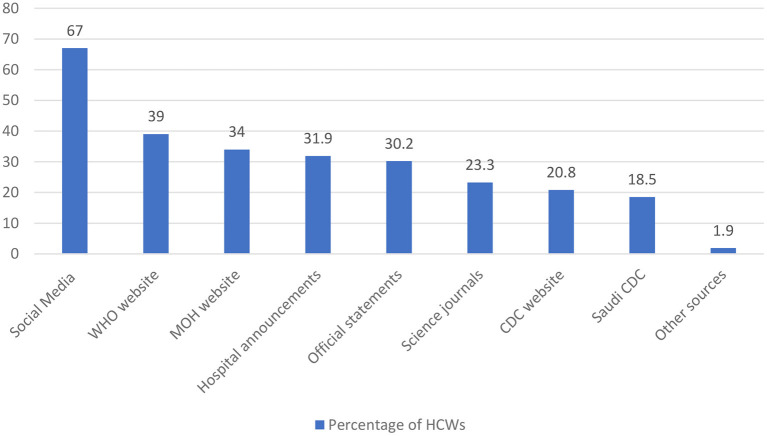
HCWs' sources of information about the B.1.1.7 SARS-CoV-2 mutant variant.

HCWs' sources of information about the mutant variant of COVID-19 are shown in Figure 1. The most common source of information was social media networks (67.0%), followed by the World Health Organization (WHO) website, Saudi Ministry of Health (MOH) website, and hospital or official announcements, while the U.S. Centers for Disease Control and Prevention (CDC) website was only used by 20.8 % of HCWs. HCWs' awareness of and knowledge about the new COVID-19 mutation was measured using eight questions (Table 3). The overall mean awareness score was 3.76 (SD: 1.23) out of 8.00.

**Table 3:** Indicators for HCWs' awareness of/knowledge about the COVID-19 pandemic and its mutations.

**Statements about Covid-19 mutation (correct answer)**	**Incorrectly answered**	**Correctly answered**
	***n* (%)**	***n* (%)**
This B.1.1.7 variant was first described in the UK (True).	144 (13.6)	914 (86.4)
The COVID virus mutation is expected (True).	277 (26.2)	781 (73.8)
This new COVID mutation is more contagious (True).	249 (23.5)	809 (76.5)
This new COVID mutation is causing a more severe disease (False).	734 (78.8)	224 (21.2)
The available COVID vaccines will be effective for new virus mutations (True).	510 (48.2)	548 (51.8)
The mutagenic virus's impact on the respiratory system is worse than the original COVID-19 (False).	870 (82.2)	188 (17.8)
To the best of your knowledge, the mutation may/will result in another wave of the pandemic (True; uncertain considered true).	673 (63.6)	385 (36.4)
The appearance of mutagenic viruses is a sign that herd immunity is occurring (False).	932 (88.1)	126 (11.9)

Most HCWs (86.4%) were aware that the new variant was first described in the U.K., 73.8% reported that the mutation is an expected evolutionary phenomenon, and 76.5% were aware that it is more contagious. However, the majority of participants believed that the new mutation would cause a more severe disease and likely have a greater negative impact on the respiratory system. In addition, most HCWs thought that the emergence of this mutation was a sign that herd immunity is occurring.

### International Travel Worries Among HCWs Due to the Emergence of Mutant COVID-19 Strains

**Figure 2:**
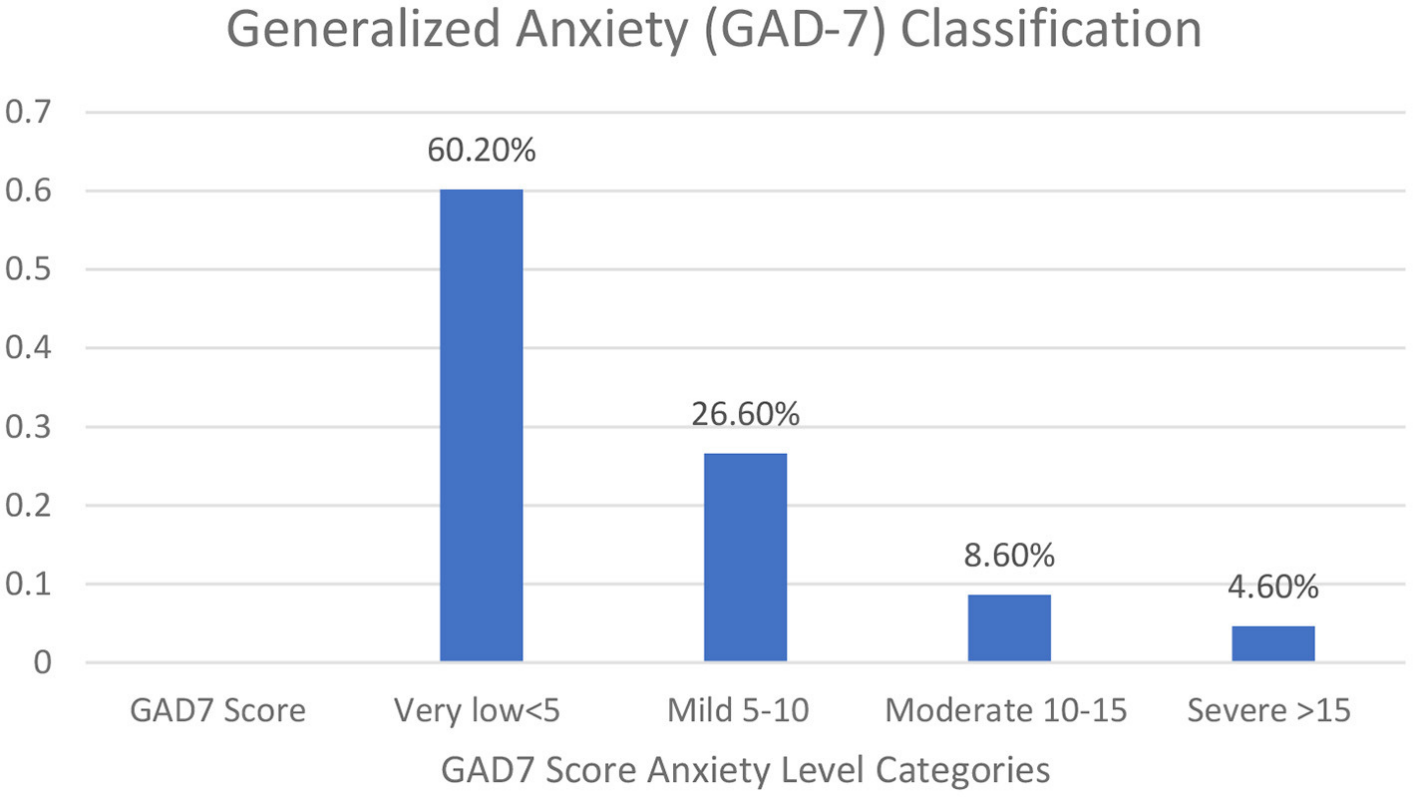
HCWs' generalized anxiety disorder assessment (GAD-7) score.

The majority of participants were expatriates living and working in Saudi Arabia. We measured HCWs' worry levels regarding international travel. In addition, we evaluated the degree of HCW anxiety using the General Anxiety Disorder (GAD−7) scale which showed a mean score (SD) of 5 (± 5) for the whole group. Figure 2 showed the distribution of the degree of anxiety among HCWs.

A bivariate analysis was conducted to illustrate the association between HCWs' characteristics and their perceived levels of worry about traveling abroad due to the emergence of the new mutation (Table 4). Female HCWs (mean = 3.36, *P* < 0.001) and those over 50 years of age (mean = 3.55, *P* = 0.024) were significantly more worried about international travel than males and HCWs under 50, respectively. In addition, expatriate HCWs had a higher level of worry regarding travel compared to native Saudi HCWs (*P* < 0.001).

**Table 4:** Bivariate analysis of HCWs' characteristics and perceived worry regarding traveling abroad due to the emergence of the mutant COVID-19 strain (*N* = 1,058).

**Characteristic**	**Mean (SD) of those who expressed concern (Q29)**	**Test statistic**	***p*-value**
**Sex**			
Male	2.96 (1.19)	t(1,056) = 5.23	<0.001
Female	3.36 (1.15)		
**Age**			
20–30 years	3.11 (1.17)	f(3,1054) = 3.17	0.024
31–40 years	3.25 (1.14)		
41–50 years	3.17 (1.22)		
≥ 51 years	3.55 (1.20)		
**Marital status**			
Single	3.21 (1.20)	t(1,056) = 0.22	0.828
Married	3.23 (1.17)		
**Nationality**			
Expatriate	3.32 (1.14)	t(1,056) = 4.01	<0.001
Saudi	3.01 (1.23)		
**Clinical Role**			
Consultant	3.01 (1.21)	f(4,1053) = 4.64	0.001
Assistant consultant/fellow	3.31 (1.15)		
Resident/registrar	2.99 (1.25)		
Nurse	3.34 (1.14)		
Intern/medical student	3.24 (1.18)		
**Hospital ward**			
ICU	3.25 (1.14)	f(6,1051) = 0.86	0.522
ER	3.25 (1.20)		
OR	3.27 (1.16)		
Isolation ward	3.00 (1.24)		
General ward	3.14 (1.26)		
OPD	3.31 (1.10)		
Non-clinical area	3.27 (1.25)		
**Hospital sector**			
Private	3.32 (1.16)	f(2,1055) = 0.70	0.510
Public/governmental	3.22 (1.20)		
University hospital	3.20 (1.16)		
**Hospital specialty**			
Primary healthcare center	3.37 (1.20)	f(32,1055) = 1.21	0.299
Secondary care hospital	3.16 (1.15)		
Tertiary hospital	3.22 (1.18)		
**Had contact with persons (any) infected with COVID-19**			
No	3.28 (1.20)	t(1,058) = 0.32	0.420
Yes	3.21 (1.17)		
**Have you been previously diagnosed with PCR-positive COVID?**			
No	3.20 (1.18)	t(1,058) = 1.95	0.051
Yes	3.45 (1.16)		
**Have you traveled abroad in the last 3 months?**			
No	3.24 (1.17)	t(1,058) = 2.10	0.037
Yes	2.92 (1.31)		
**Did you receive or register to get the COVID-19 vaccine?**			
Yes: I have received the first dose of the COVID vaccine	2.85 (1.30)	f(2, 2055) = 8.12	<0.001
Yes: I have registered but have not yet received it	3.20 (1.11)		
No	3.30 (1.17)		
**Use of social media for information**			
No	3.10 (1.20)	t(1,056) = 3.36	0.001
Yes	3.31 (1.60)		

Nurses and interns/medical students were more worried than those assigned to other clinical roles (*P* = 0.001). Additionally, those who reported they had not traveled in the previous 3 months and those who had not received or registered for the COVID-19 vaccine were also significantly more worried than those who had traveled or received the vaccine (*P* = 0.037, *P* < 0.001, respectively). Interestingly, those HCWs who used social media as a source of information were significantly more worried about traveling abroad due to the emergence of the new mutant strain.

Further, multivariate linear regression analyses (Table 5) were used to predict respondents' characteristics based on their level of worry associated with traveling abroad. This revealed a significant correlation for HCWs' level of travel worry with older age, female sex, higher GAD-7 scores, abstinence from traveling abroad in the previous 3 months, and the belief that HCWs should apply tighter infection control measures due to the emergence of mutant strains. However, no significant correlation was found between HCWs' level of travel worry and their status as having received or registered to receive the vaccine. In addition, the analysis model indicated that people who had traveled abroad recently had a significantly lower mean level of worry regarding international travel outside the UK (β = −0.437, *p* < 0.001).

**Table 5:** Multivariate linear regression analysis of HCWs' level of worry regarding traveling abroad (*N* = 1,058).

**Characteristic**	**Unstandardized β Coefficient**	**95% CI for** **β** **coefficient**		***p*-value**
		**Lower Bound**	**Upper Bound**	
Constant	1.483	0.922	2.043	<0.001
Sex = Female	0.208	0.037	0.379	0.017
Age (years)	0.018	0.010	0.026	<0.001
Nationality = Saudi	0.049	−0.121	0.219	0.575
Hospital setting	−0.070	−0.165	0.024	0.144
Confirmed as previously having COVID-19	0.143	−0.086	0.372	0.220
GAD-7 score	0.065	0.052	0.078	<0.001
Mean score for awareness of mutant virus (min = 0, max = 8 points)	−0.035	−0.092	0.022	0.227
Had previous contact with COVID-19 infected person(s)	−0.142	−0.301	0.017	0.079
Had traveled abroad in the last 3 months	−0.437	−0.723	−0.152	<0.001
Had registered/received the COVID-19 vaccine	−0.058	−0.208	0.092	0.448
Believes HCWs should apply tighter infection control measures	0.230	0.165	0.296	<0.001
Used social media for information on COVID-19/mutated viruses	0.129	−0.015	0.272	0.080

Furthermore, a bivariate Pearson's correlation test was conducted and showed that HCWs' worry about international travel correlated significantly and positively with their GAD-7 scores (*r* = 0.310, *p* < 0.010) and perceived importance of applying stricter infection control measures (*r* = 0.256, *p* < 0.010).

## Discussion

In this first reported survey on SARS-CoV-2 B.1.1.7 lineage-related perceptions and travel worry among HCWs, most respondents were aware of the emergence of the variant and had significant anxiety regarding traveling to affected countries, especially to the UK. At the time of our study, there were no published data on B.1.1.7 lineage from the region. Later on, in one study from Jordan, the B.1.1.7 variant was isolated from 36 (6.2%) of studied isolates (15). By Feb 2021, 13 countries in the Middle East reported B.1.1.7 variants (16).

Females and nurses expressed the highest degree of worry about travel, and most of our study participants were nurses (59.0%), with two-thirds being expatriates. Our respondents, therefore, are comparable to the HCWs structure in Saudi Arabia, where physiciansccomprise 36.2 %), and nurses (63.8%) (17). Saudi Arabia's health care system workforce relies heavily on expatriates, specifically female nurses, as only one-third of the nursing workforce is comprised of Saudis (18). That fact shed light on these HCWs being expatriate who usually resides in housing compounds in shared rooms and facilities (19) that can be hazardous in terms of transmission of the disease among themselves and within the health care system, a previous MERS-CoV hospital outbreak in Saudi Arabia was in part related to such accommodations (20). Travel needs could explain why expatriate female nurses, who make up most survey respondents, have such a high degree of concern about international travel, adding to that the fact many of these expatriate HCWs have been stranded abroad during the pandemic due to travel restrictions and the emergence of the variant strain with potential tighter restrictions on traveling to be applied or reapplied in addition to high travel anxiety among those HCW all potentially might endanger our health care system manpower. These findings and previous experiences with MERS-CoV and our findings should alert public health officials to reevaluate their long-term strategy for training and empowering local frontline HCWs.

On the other hand, just recently, HCWs in Saudi Arabia were exempted from the travel bans. In our survey, a few HCWs indicated that they had traveled to countries with potential cases of the new variant in recent months, despite quarantine measures on symptomatic returning travelers. HCWs who return from international travel could pose a potential risk of introducing emerging variants (21). Almost two-thirds of participants had dealt with COVID-19 infected patients, 23.0% had a family member who had been diagnosed with the disease, and about 10.0% had been infected themselves. These results are similar to a previous cross-sectional survey of nearly 1,500 HCWs in Saudi Arabia, which found that 12.8% of HCWs had been infected (13). In that same report, two-thirds of participants were willing to receive an authorized COVID-19 vaccine when available. However, our findings of the actual implementation of the vaccine contradict this as 66% didn't register or receive the vaccine even though of our findings of moderate level of anxiety to travel abroad among surveyed HCWs which should intuitively motivate any person to take the vaccinedespite the vaccine being authorized and available free of charge in Saudi Arabia. These findings should also alert public health officials to introduce targeted vaccine campaigns among HCWs.

Most participants were aware of the new variant reported, with more than one-third indicating confidence in their knowledge. However, only one-fifth were aware that it may cause a false negative PCR result. Recently, the U.S. Food and Drug Administration (21) identified three different molecular techniques that may be affected by the variant and recommended that HCWs be aware of the different genetic variants of SARS-CoV-2 that may affect test results depending on the type of molecular test used to diagnose a patient. Of notion, the majority of the real-time PCR Kits used routinely in Saudi Arabia for detection of SARS-CoV-2 do not contain the S gene; thus, the effect of the S gene dropout pattern will not be significant. Negative results should be considered in combination with a clinical evaluation (21).

Our study showed that 86.0% of HCWs were aware that the new variant was first described in the U.K. and that many countries have subsequently reported cases. This highlights the importance of HCWs and infection control authorities keeping up to date about those countries when assessing returning travelers. With the rapidly changing picture of variants circulating in each district or city, this may be different inside each country as well (22).

Regarding the behavior of the mutant strain, most of our HCWs knew that it is more contagious, and they incorrectly expected it to cause a more severe disease than the original strain. The new variant has been reported to be 56% more infectious (23). When asked whether the current COVID-19 vaccine would prevent infection, this was met with uncertainty which might have partially affected their low uptake of the vaccine. It is not yet scientifically proven whether any type of vaccination will be effective in preventing infection from emerging variants. *In-vitro* studies have shown that the BNT162b2 vaccine neutralizes SARS-CoV-2 viruses carrying N501Y mutations, but more clinical data are still needed (24).

In the current study, 67.0% of the respondents acquired their information from social media. Social media has played an important role in information accuracy as well as spreading misleading information during the COVID-19 pandemic (25). In a previous study from the KSA, 44.1% of participants used a social media platform such as Twitter (25). In our study, 73.0% of the respondents used either the WHO or MOH websites for information. This is an important finding that may direct plans for the dissemination of information about the pandemic.

It is interesting to note that in the bivariate analysis, HCWs' perceived level of travel worry was significantly associated with their use of social media as a source of information. This association may be related to the high degree of fear that tends to be reflected in the media. However, a study from the U.S. found a decreased level of concern about COVID-19 among individuals who used news outlets such as CNN or Fox News as their primary source of information (26).

Other factors that were significantly associated with HCWs' decreased level of travel worry were being male, older age, and being a Saudi national. Several factors have been shown to contribute to people's general levels of anxiety about COVID-19. A review study from China and India that included 18 studies with a total of 79,664 participants showed that the level of stress among the general population regarding COVID-19 was associated with having patient contact and being of the female gender (27). In addition, our study showed increased levels of travel worry among nurses and assistant consultants/fellows; however, it is important to keep in mind that the level of anxiety about COVID-19 is also a factor of time, as one study showed variable anxiety levels as the pandemic has evolved (28). It is expected that individuals who worry about additional lockdowns would also be concerned about travel, as shown by Lee et al. (29). It is interesting to note that in our study, participants who answered that the mutation may cause a false negative PCR result were more worried about travel. The SARS-CoV-2 mutation may or may not result in a false negative PCR result based on the location of the mutation and the molecular test being used (30).

COVID-19 has been associated with significant anxiety among HCWs in Saudi Arabia (12, 31). Our descriptive Pearson's bivariate correlations analysis showed that there was a significant correlation between worry regarding travel, the perceived importance of infection control, and generalized anxiety. This is an important finding, as a previous study found that increased resilience scores is a factor that reduces the rate of anxiety related to COVID-19 (32).

However, despite an overall low to mild GAD-7 scores, we found a link between advanced age and worry regarding travel which seems expected, as older people have more health concerns and are more susceptible to severe forms of infection and a higher fatality rate (33). To add to this, HCWs have a higher risk of catching COVID-19, with an estimated risk of three to 5-fold greater than that of the general population (34). The literature has also shown that older HCWs are more affected by the Pandemic. For example, the median age of HCWs who have died due to COVID-19 in China is 55 years (35). As a result, the U.S. Department of Labor recommends recognizing older age as an individual risk factor and addressing it when planning for pandemics (36). P This background could explain why HCWs in this study recommended applying tighter infection control measures than those currently in place. It also may speak to their recent experiences as frontline workers during the Pandemic, as most of our respondents were registered nurses.

HCWs who had abstained from travel recently were more worried about traveling abroad. Other studies have observed a correlation between threat severity and susceptibility to the virus, which has caused travel fear and resulted in travel restrictions due to the COVID-19 outbreak (37). Studies have reported that similar post-disaster travel behaviors were influenced by risk perceptions and motivations (38, 39).

We used a self-reported anxiety questionnaire (GAD-7) that is designed to assess the participants' mental health status during the previous 2 weeks, which is well-suited to the situation of emerging news about this mutation. The GAD-7 questionnaire inquires about the overall degree to which the respondent has felt nervous, with free-floating anxiety themes (40). A previous study showed that HCWs had a higher prevalence of anxiety at baseline prior to the Pandemic (41). Similarly, numerous national studies have reported an increased level of anxiety symptoms in HCWs during the COVID-19 Pandemic (42–44). To understand this finding, we hypothesize our study likely included HCWs with high baseline anxiety levels, whose anxiety levels are now increased even more due to the SARS-CoV-2 new mutation.

HCWs' travel worries due to the new SARS-CoV-2 genetic variants should be addressed, especially because HCWs are more prone to developing mental health problems such as anxiety, depression, and substance abuse during pandemics (45, 46). Therefore, screening and supporting expatriate or traveling HCWs could improve their mental well-being, as well as advising against HCWs' travel except for “really needed” until the pandemic crisis is over.

### Study Limitations and Strengths

This work is among the first research projects to explore travel worries among HCWs regarding the travel worries and restrictions caused by new genetic variants of the SARS-CoV-2 virus. However, there are some limitations to this study. We have used convenience sampling, potentially limiting the representation of the study's sample. Responses were predominantly from COVID-19 frontline physicians and nurses that did not include some categories of HCWs, such as dentists and laboratory workers, which resulted in possible selection bias. We have used an online questionnaire, potentially introducing voluntary response and non-response bias since motivated and internet literate respondents would complete the survey. This could further reduce the generalizability of our findings.

However, our sampling technique and the cross-sectional design are employed based on the study's objective during the ongoing Pandemic. For our study, it was to quickly collect data to generate hypotheses, signifying that convenience sampling would be satisfactory. This is especially important with the limited published studies on travel worries among HCWs regarding the travel restrictions caused by new genetic variants of the SARS-CoV-2 virus. Nevertheless, the study findings, including associations, should be interpreted with caution. We believe that potential future research would build on our findings with other approaches to data collection and sampling techniques to ensure a more representative sample.

## Conclusion

Most HCWs were aware of the emergence of the SARS-CoV-2 B.1.1.7 variant and expressed substantial travel worries in relation to it. The levels of travel worries were greater among expatriate, female nurse HCWs, those who used social media as their main source of information, who had not registered for the COVID-19 vaccine, and who had high GAD-7 scores. Such high levels of worry, especially among expatriate HCWs, need to be addressed urgently in order to maintain our workforce capacity to keep facing the ongoing Pandemic. In addition, public health authorities' utilization of official social media platforms could improve the dissemination of accurate information among HCWs regarding the virus's evolving mutations. Targeted vaccine campaigns are warranted primarily to ensure HCWs uptake of the vaccine being the frontline in the current Pandemic and to make them aware of the efficacy of COVID-19 vaccines toward the genetic variants of SARS-CoV-2.

## Data Availability Statement

The original contributions presented in the study are included in the article/supplementary material, further inquiries can be directed to the corresponding authors.

## Ethics Statement

The studies involving human participants were reviewed and approved by IRB, King Saud University, Riyadh, Saudi Arabia. Written informed consent for participation was not required for this study in accordance with the national legislation and the institutional requirements.

## Author Contributions

M-HT, MB, JA-T, FAlj, ANA, AA-E, and RH conceptualized the study, analyzed the data, and wrote the manuscript. BS, FAls, AAlh, KA, AAla, SA, and RT contributed to the study design, collected, analyzed, and interpreted data, and edited the manuscript. NA contribution to the study design and interpretation and edited the manuscript. FAlz, AJ, and SE interpreted the data and finalized the manuscript. All authors contributed to the article and approved the submitted version.

## Conflict of Interest

The authors declare that the research was conducted in the absence of any commercial or financial relationships that could be construed as a potential conflict of interest.

## Abbreviations

CDC: Centers for Disease Control and Prevention
COVID-19: Coronavirus disease 2019
GAD-7: Generalized Anxiety Disorder Assessment
SARS-CoV-2: Severe acute respiratory syndrome coronavirus 2
WHO: World Health Organization.
